# Association of *HOTAIR* gene rs920778 (C > T) and rs4759314 (A > G) polymorphism with breast cancer in Egyptian women

**DOI:** 10.1007/s11033-023-08725-6

**Published:** 2023-09-30

**Authors:** Nahla Anber, Mohammad M. Tarabay, Rehab Elmougy, Marwa Ahmed Abdel-Dayem, Ehab Yones Elbendary

**Affiliations:** 1 Emergency Hospital, Faculty of Medicine, Mansoura University, Mansoura, Egypt; 2 Health Insurance Organization (HIO), Mansoura, Mansoura, 35511 Egypt; 3 Biochemistry Division, Chemistry Department, Faculty of Science, Mansoura University, Mansoura, Egypt; 4 Pharmacology and Toxicology Department, Faculty of Pharmacy, Horus University, New Damietta City, Egypt; 5 Clinical Nutrition Department, College of Applied Medical Sciences, Jazan University, Jazan, Kingdom of Saudi Arabia

**Keywords:** *HOTAIR* polymorphisms, rs920778, rs4759314, Breast cancer

## Abstract

**Background:**

Hox transcript antisense RNA (*HOTAIR*) is considered an oncogene associated with the initiation and progression of many malignancies. Previous studies have examined the connection between *HOTAIR* SNPs rs4759314 and rs920778 for breast cancer (BC), getting variable results in multiple ethnicities. Therefore, this study was designed to evaluate the connection between these two SNPs and disease vulnerability, clinic-laboratory, and hormonal parameters, featuring status associations with the BC risk in an Egyptian woman sample.

**Methods and results:**

In this case-control study, DNA was taken from the blood of 100 BC patients and 100 unrelated healthy Egyptian females. The characterization of rs4759314 was genotyped using the T-ARMS-PCR method and rs920778 using the SNP-RFLP technique for all participants. The frequency of the rs4759314 A > G variation revealed a statistically significant increase in BC risk with dominant (p = 0.013, OR = 1.592, 95% Cl = 1.105–2.293), co-dominant (p = 0.006, OR = 2.314, 95%Cl = 1.278–4.191) and overdominant (p = 0.002, OR = 2.571, 95% Cl = 1.430–4.624) genetic models. On the other hand, the rs920778 C > T polymorphism was not significantly associated with BC. ER/PR positivity with *HER2* negativity was significantly associated with the AA genotype compared to the AG genotype. Otherwise, no significant associations between the two SNPs and clinical stage or hormonal features could be found. In conclusion, the rs4759314 A > G SNP in the *HOTAIR* gene is strongly associated with BC, which might warrant its determination among affected families for prevention and early treatment.

## Introduction

Breast cancer (BC) is the most common cancer among women; however, it is the main reason for death from cancer in women worldwide [1]. BC is the most prevalent cancer globally, with around 8 million women surviving in 2020 having been diagnosed in the preceding five years [2, 3]. Each year, over 22,000 new cases are identified. Each year in Egypt, 33% of all female cancer cases are detected; however, this proportion is predicted to climb dramatically in the coming years due to the expanding population [4, 5].

BC is a complicated illness, including environmental and genetic variables. Single nucleotide polymorphisms (SNPs) are often employed to predict disease risk, clinical outcome, and prognosis [6].

Long non-coding RNAs (lncRNA) have garnered more attention during the past few years, including SNPs that may alter cancer and other human disease risks. LncRNA is characterized as transcripts that are longer than 200 nucleotides and have no protein-coding potential [7, 8]. SNPs, copy-number changes, and non-coding genome mutations can greatly impact lncRNA production [9, 10].

*HOTAIR* as a lncRNA results from the *HOXC* gene, whose significance in the invasion and development of several types of tumors is well established [11, 12]. Many scientists have investigated the relationship between cancer prognosis and *HOTAIR* expression. However, they revealed that *HOTAIR* is suspected to be a cancer-causing oncogene. Its genetic variations increase intronic activity and enhance *HOTAIR* expression in specific cancer cells [13–15]. Two *HOTAIR* SNPs, rs920778 C > T and rs4759314 A > G, were selected to test their association with breast cancer susceptibility because they have previously been linked to elevating cancer risk.

The *HOTAIR* rs920778 polymorphism is in the *HOTAIR* gene intron 2 and results from the substitution of cytosine for thymine (C→T). The *HOTAIR* gene’s intron 2 contains a new intronic enhancer that is the home to the *HOTAIR* rs920778 polymorphism, which causes T allele carriers to express *HOTAIR* more frequently [16]. The polymorphism of rs4759314 (A > G) results from the replacement of adenine with guanine (A→G). Furthermore, it was found that the GG genotype can enhance *HOTAIR* expression by boosting *HOXC* promoter activity [11].

In carcinomas, the human epidermal growth factor receptor 2 oncogene (*HER2*) encodes a protein that activates cell signaling networks that influence various malignant cells. Through a complementary target location in *HOTAIR*’s final exon, *HOTAIR* works as a competitive endogenous RNA to negatively control miR-331-3p, preventing miR-331-3p-mediated suppression of the oncogene *HER2* [17]. Subsequently, Our case-control study’s major purpose is to evaluate the connection between *HOTAIR* polymorphisms (rs4759314 and rs920778) and disease vulnerability, clinic-laboratory parameters, and hormonal parameters featuring status association with the BC risk in an Egyptian woman sample.

## Patients and methods

### Studied participant

Our research was performed in line with the principles of the Declaration of Helsinki. Approval was granted by the Egyptian Medical Research Ethics Committee, Faculty of Medicine, Mansoura University, Egypt (IRP Cod (R.22.06.1746)). Before enrolling in this study, all female participants provided a completed permission form. The study techniques were conducted in accordance with the approved protocols.

Our study considered a case-control study where potentially eligible participant patients were 250 cases diagnosed with BC and recruited between September 2022 and January 2023 from outpatient clinics of Mansoura University’s oncology center, Mansoura University Hospital, Egypt. Of the 250 cases, only 100 newly diagnosed cases underwent the study. In comparison, 150 cases were excluded from the research, including those with a history of cancer, metastasis to other sites, radiation exposure, autoimmune disease, immunological syndromes, or the use of any medicine, including those for hormonal, chemical, or radiological reasons. As a control group, 100 age-matched, seemingly healthy females with no history of health issues, typical routine checkups, and comparable socioeconomic variables. BC diagnosis was validated by histopathological examination for tumor biopsies; however, pathologists conducted them for tumor staging [18] and grading [19] evaluation. The BC prognostic biomarkers (*HER2*, estrogen, and progesterone receptors (ER/PR)) were examined *via* immunohistochemical methods [20].

### Sample collection

Blood samples from people under examination (5 ml) were separated into two portions; some of the blood was deposited in vacutainer tubes without additives for tumor markers and biochemical evaluation. The remainder was drawn into vacutainer tubes containing the anticoagulant EDTA for hematological and genetic examination.

### Evaluation of tumor markers, biochemical, and hematological assessment

Using enzyme-linked immunosorbent assay (ELIZA) kits, cancer antigen 15–3 (CA 15–3) was measured. A hematological cell analyzer (CELL-DYN 3700 SL, Abbott Diagnostics, USA) was used to measure hematological parameters, including leukocytes, lymphocyte count, erythrocytes, hemoglobin level, and platelet count. Biochemical estimations of serum transaminase enzymes (aspartate transaminase [19] and alanine transaminase [20], alkaline phosphatase (ALP), total bilirubin, albumin, uric acid, and creatinine were conducted using a Cobas c501, Roche Diagnostics Mannheim, Germany, fully automated biochemical analyzer.

### Detection of gene polymorphisms (genotyping)

#### DNA extraction

Using the Qiagen DNA purification (Valencia, CA) kit, genomic DNA was obtained from peripheral blood according to the manufacturer’s recommendations.

#### PCR amplification and genetic typing assay

For genotyping the rs920778 polymorphism, the restriction fragment length polymorphism (PCR-RFLP) technique was utilized (Ana XavierMagalhães et al. 2017). The PCR protocol was executed using (Applied Biosystems, Foster City, CA), a thermal cycler. Briefly, rs920778 is amplified in a volume of 22 μl, including a DNA template (4 μl), forward & reverse primers (4 μl), and a PCR master mix (10 μl, Thermo Scientific). Adjustments were made to the reaction conditions, beginning with a denaturation stage for 5 min at 95 °C, followed by 35 cycles of 95 °C for 60 s, 58 °C for 60 s, and 72 °C for 60 s, and a final step at 72 °C for 10 min to allow for the extension of all PCR fragments. Consequently, a PCR amplification fragment of 234 bp was produced using the primers forward: 5′-TTA CAG CTT AAA TGT CTG AAT GTT CC, and reverse: 5′-TAT GCG CTT TGC TTC CAG.

For rs920778, an *MSPI* (Thermo Fisher Scientific) restriction enzyme was used to digest the PCR products after the 234 bp. The resulting assimilation fragments were electrophoresed using agarose gel (2%) and dyed with ethidium bromide to make them easier to see under UV light. Finally, these fragments were identified as follows: the homozygous wild type (CC) generated two fragments at 218 bp and 26 bp, the heterozygous (CT) genotype generated three fragments at 234 bp, 218 bp, and 26 bp, whereas the homozygous (TT) genotype produced just one fragment at 234 bp.

The rs4759314 was genotyped using a tetra-primer amplification refractory mutation system with PCR (T-ARMS-PCR). The thermal cycler of PCR denaturation temperature at 94^o^C for 4 min, followed by 35 cycle denaturation at 94^O^C for 45 s, annealing temperature of 54.5^O^C for 45 s, extension temperature of 72^O^C for 55 s, and final extension of 72^O^C for 10 min. The primer sequence was as follows:

reverse outer primer (5’- 3’) CCAAGGTAGGGAAGTCTCTATTTCTCTG;

forward outer primer (5’- 3’) AAACCATATCCTGACAGAAGCCAAATAC;

reverse inner primer (G allele) TTATCACGTTTTATTAACTTGCATCCTCC;

forward inner primer (A allele) GCATGGAAGAGATATAAACAGGCGAA.

The resultant assimilation fragments were electrophoresed on a 2% agarose gel and dyed with ethidium bromide to be visible by UV light. The resultant fragment size was 24 bp by outside primers, 121 bp for the G allele, and 181 bp for the A allele.

### Sample size and statistical analysis

The sample size was calculated using the GAS Power Calculator, 2017. This calculation was based on a previous study by Lv et al. [21], who showed an elevated frequency of the G allele for rs920778 in patients with breast cancer compared to the control group, considering the expected odds ratio of 1.7, prevalence of breast cancer of 13%, disease allele frequency of 23%, a minimal sample size of 100 for cases and 100 for controls is required with a power of 80% and a significance level of 5%.

The data were modified, coded, tabulated, and uploaded to a computer using IBM’s 2017-released Statistical Software for Social Science, IBM SPSS version 25.0 for Windows (Armonk, New York: IBM Corporation, 2005). The t-test and Mann-Whitney test were used to compare the means of two groups, while Kruskal-Wallis tests and one-way analysis of variance (ANOVA) were used to compare the means of more than two groups. Deviations from Hardy–Weinberg equilibrium expectations among control groups were assessed to be in equilibrium using the chi-squared test. The odds ratio and 95% confidence intervals were obtained using logistic regression analysis. All reported p-values were two-tailed, and a p-value of 0.05 was statistically significant.

## Results

### The baseline characteristics, biochemical assessment, and clinicopathological variables of the study population

This study was performed on 100 female BC with a mean age of 48 ± 10.6 years. BC cases were significantly associated with a positive family history. Tumor marker assessment identified significantly higher CA15.3 serum levels in BC patients (24.4 U/ml) when compared to the control (21.185 U/ml) (p = 0.001), while no significant differences in hematological and biochemical markers were identified between patients and controls (Table 1). According to the BC stage, 87/100 (87%) cases were localized (non-metastatic) (stages 1 and 2), while 13/100 (13%) patients represented metastatic cases (stages 3 and 4). On the other hand, according to the BC grade, 52/100 (52%) were grade 2. According to hormonal features, 66% of cases were ER/PR positive, 77% were *HER2* negative, and 46% were ER/PR negative-*HER2* positive (Table 2).

**Table 1:** Demographic and laboratory data of the studied subjects

		Control	Cases	p-value
		n = 100	n = 100	
Age (years)		47 ± 7.5	48.3 ± 10.6	0.306
Age	< 40	25(25%)	26(26%)	0.871
	> 40	75(75%)	74(74%)	
	< 45	45(45%)	45(45%)	1
	> 45	55(55%)	55(55%)	
FH	Negative	0(0%)	64(64%)	< 0.001
	Positive	0(0%)	36(36%)	
ALT (U/L)	Median (range)	22.21(7.5–52.2)	19(3-199.9)	0.137
AST (U/L)	Median (range)	26.51(6.4–49.1)	23(9-147.1)	0.591
T. bilirubin (mg/dL)	Median (range)	0.495(0.2-1)	0.5(0.2–1.2)	0.933
ALP (U/L)	Median (range)	153.5(66–288)	137(66–355)	0.096
SrCr (mg/dL)	Median (range)	0.74(0.2–9.6)	0.8(0.4–3.2)	0.108
Uric acid (mg/dL)	Median (range)	4.1(2.1–6.9)	4.2(2.2–11.5)	0.186
CEA (μg/L)	Median (range)	2.775(0.5-5)	2.325(0.5–33.5)	0.658
CA15.3 (U/mL)	Median (range)	21.185(5.3–31.6)	24(8.6–87)	< 0.001
Albumin (mg/dL)	Mean ± SD	3.9 ± 0.6	4.1 ± 0.5	0.071
WBCS (X10^9^]/L)	Mean ± SD	6.6 ± 1.3	7.4 ± 2.1	0.134
RBCS (cells/mcL)	Mean ± SD	4.6 ± 0.7	4.3 ± 0.6	0.101
Hb (g/dL)	Mean ± SD	11.9 ± 1.1	11.5 ± 1.8	0.276
PLT (X10^9^/L)	Mean ± SD	253.9 ± 54	284.1 ± 94.7	0.227

Fisher’s exact test and Mann Whitney- U test.

FH, family history; ALT, alanine transaminase; AST, aspartate transaminase; T. bilirubin, Total bilirubin; ALP, alkaline phosphatase; SrCr, serum creatinine; CEA, carcinoembryonic antigen; CA15.3, cancer antigen 15 − 3; WBCS, white blood cells; RBCS, red blood cells; HB, Hemoglobin; PLT, platelet.

**Table 2:** Clinico-pathological tumor features among cases

		Cases
		**n = 100**
Stages	S1 + S2	87(87%)
	S3 + S4	13(13%)
	S1	23(23%)
	S2	64(64%)
	S3	9(9%)
	S4	4(4%)
Grades	G1 + G2	74(74%)
	G3	26(26%)
	G1	22(22%)
	G2	52(52%)
	G3	26(26%)
ER/PR	-ve	34(34%)
	+ve	66(66%)
*HER2*	-ve	77(77%)
	+ve	23(23%)
ER/PR-*HER2*	Triple –ve	31(31%)
	-ve/ +ve	46(46%)
	+ve/ -ve	3(3%)
	Triple + ve	20(20%)

ER/PR; estrogen/progesterone, *HER2*; human epidermal growth factor receptor 2, -ve; negative, +ve; positive.

### Genotype and allelic distribution in studied groups and risk for BC

**Fig. 1:**
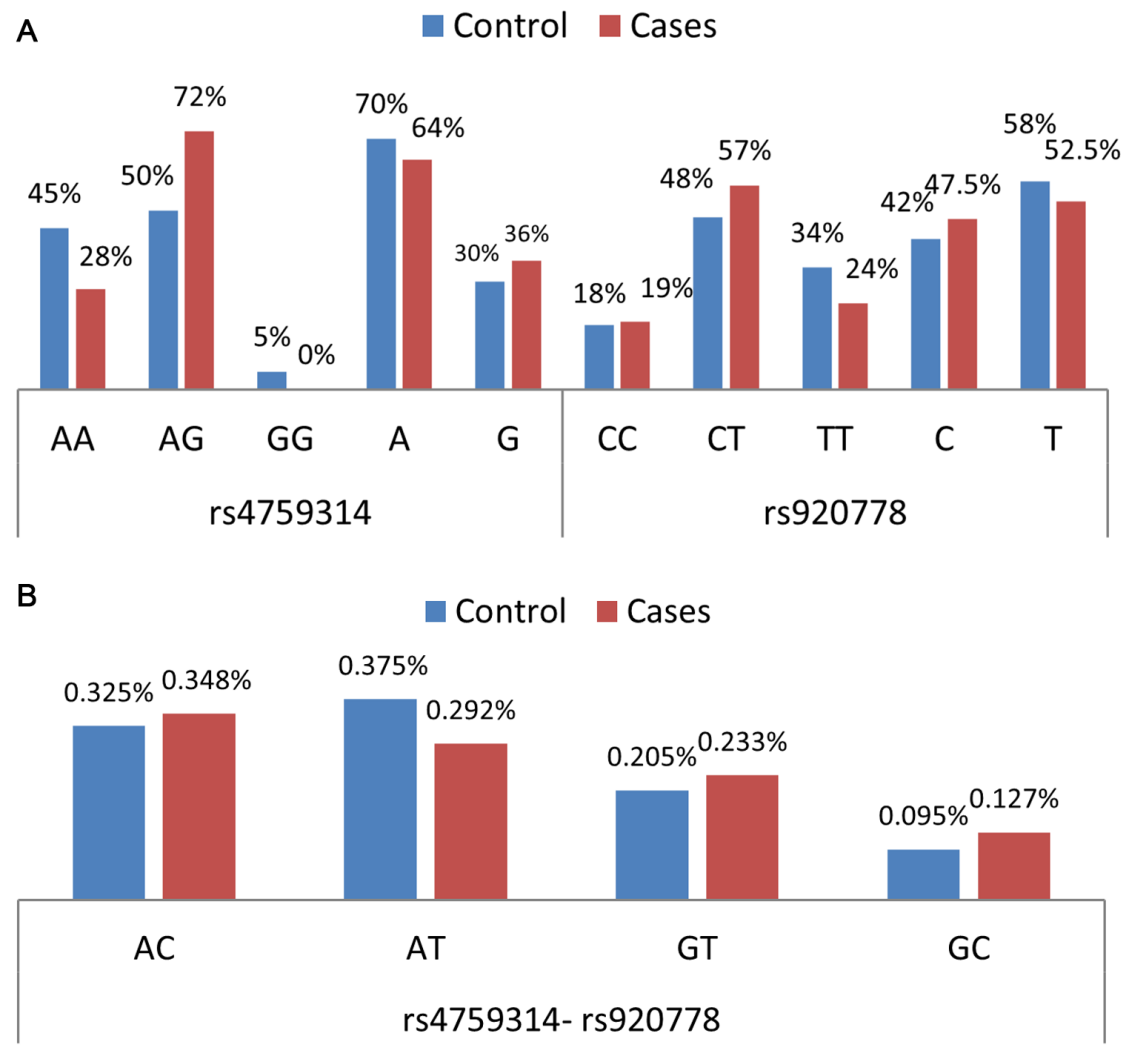
Genotypes and haplotypes distribution. (**A**) Frequency of rs4759314 and rs920778 genotypes among studied groups. (**B**) Frequency of rs4759314- rs920778 haplotypes among studied groups

The present study revealed two alleles of rs4759314: allele A (70% control, 64% cases; 181 bp) and allele G (30% control, 36% cases; 121 bp). Further, the results explored three genotypes, including AA, AG, and GG, with a low frequency of the GG genotype among patients (0%) and controls (5%) (Table 3; Fig. 1a). In addition, the result revealed two alleles of rs920778, including allele C (42% control, 47.5% cases; 218 bp, and 26 bp) and allele T (58% control, 52.5% cases, and 234 bp); however, the result showed three genotypes: CC, CT, and TT (Table 3; Fig. 1a).

The connection between rs4759314 and rs920778 SNPs and the risk of developing BC was investigated using regression analysis. rs4759314 patients had a considerably greater prevalence of the AG genotype (72% versus 50%) (p = 0.005, OR = 1.689, 95% CI = 1.168–2.441), dominant AA versus AG + GG (p = 0.013, OR = 1.592, 95% CI = 1.105–2.293), co-dominant AG versus AA (p = 0.006, OR = 2.314, 95% CI = 1.278–4.191), and over dominant AA + AG versus GG (p = 0.002, OR = 2.571, 95% CI = 1.430–4.624) compared to controls, with suggested susceptibility of BC (Table 3).

**Table 3:** Association of rs4759314 A > G genotypes and alleles with BC

			Control				Cases				p-value		OR		95% CI			
			**N**	**%**			**N**		**%**									
Genotypes	**AA**	45			45.0	28		28.0		**-**		**1**		**Reference**				
	**AG**	50			50.0	72		72.0		0.005		1.689		1.168			2.441	
	**GG**	5			5.0	0		0		1		-		-			-	
Dominant model	**AA**	45			45.0	28		28.0		**-**		**1**		**Reference**				
	**AG + GG**	55			55.0	72		72.0		0.013		1.592		1.105			2.293	
Co-dominant	**AA**	45			45.0	28		28.0		**-**		**1**		**Reference**				
	**AG vs. AA**	50			50.0	72		72.0		0.006		2.314		1.278			4.191	
	**GG vs. AA**	5			5.0	0		0		1		-		-			-	
Recessive model	**AA + AG**	95			95	100		100		**-**		**1**		**Reference**				
	**GG**	5			5.0	0		0		1		-		-		-		
Overdominant	**AA + GG**	50			50	28		28		**-**		**1**		**Reference**				
	**AG**	50			50	72		72		0.002		2.571		1.430			4.624	
Alleles	**A**	140			70.0	128		64.0		**-**		**1**		**Reference**				
	**G**	60			30.0	72		36.0		0.202		1.186		0.913			1.540	
HW	**X2**	3.628				31.641												
	p-value	0.057				< 0.001												

OR, odds ratio; CI, confidence interval; HW, Hardy Weinberg.

In contrast, the rs920778 polymorphism genotypes of patients and controls did not differ significantly (p > 0.05) across all genetic models, including the dominant and recessive models.

On the other hand, our results reported no significant difference between the case group and control regarding rs920778 polymorphism genotypes (p > 0.05) in all genetic models, including the dominant and recessive models (Table 4).

**Table 4:** Association of rs920778 C > T genotypes and alleles with BC

		Control		Cases		p-value	OR	95% CI			
		**N**	**%**	**N**	**%**						
Genotypes	**CC**	18	18.0	19	19.0	**-**	**1**	**Reference**			
	**CT**	48	48.0	57	57.0	0.758	1.077	0.673	1.722		
	**TT**	34	34.0	24	24.0	0.342	0.777	0.463	1.306		
Dominant model	**CC**	18	18.0	19	19.0	**-**	**1**	**Reference**			
	**CT + TT**	82	82.0	81	81.0	0.856	0.959	0.613		1.501	
Co-dominant	**CC**	18	18.0	19	19.0	**-**	**1**	**Reference**			
	**CT vs. CC**	48	48.0	57	57.0	0.758	1.125	0.531	2.382		
	**TT vs. CC**	34	34.0	24	24.0	0.342	0.669	0.292	1.533		
Recessive model	**CC + CT**	66	66	76	76	**-**	**1**	**Reference**			
	**TT**	34	34.0	24	24.0	0.121	0.613	0.330	1.137		
Overdominant	**CC + TT**	52	52	43	43	**-**	**1**	**Reference**			
	**CT**	48	48.0	57	57.0	0.203	1.436	0.822	2.507		
Alleles	**C**	84	42.0	95	47.5	**-**	**1**	**Reference**			
	**T**	116	58.0	105	52.5	0.269	0.870	0.679	1.114		
HW	**X2**	0.022		2.041							
	p-value	0.883		0.153							

OR, odds ratio; CI, confidence interval; HW, Hardy Weinberg; vs., versus.

### Correlation of gene polymorphism variants with laboratory, clinical, and hormonal features in a patient group

rs4759314 and rs920778 genotypes showed no significant associations with studied demographic data or lab measurements (Table 5). On the other hand, associations of studied genotypes with clinical and hormonal features revealed that ER/PR positivity with *HER2* negativity was significantly associated with AA compared to AG in the rs4759314 genotype. Otherwise, no significant associations could be found between the two SNPs and BC patients’ clinical stage, ER/PR, histological grade, or *HER2* protein expression (Table 6).

**Table 5:** Association of rs4759314 A > G and rs920778 C > T genotypes with demographic and laboratory parameters among BC cases

			rs4759314 A > G			rs920778 C > T			
			**AA**	**AG**	**p-value**	**CC**	**CT**	**TT**	**p-value**
			**n = 28**	**n = 72**		**n = 19**	**n = 57**	**n = 24**	
Age (years)		mean ± SD	49.1 ± 9.4	48 ± 11	0.619	45.4 ± 10.7	48.9 ± 9.8	49.1 ± 12.1	0.428
Age	< 40	N (%)	5(17.9%)	21(29.2%)	0.247	8(42.1%)	12(21.1%)	6(25%)	0.214
	> 40	N (%)	23(82.1%)	51(70.8%)		11(57.9%)	45(78.9%)	18(75%)	
	< 45	N (%)	10(35.7%)	35(48.6%)	0.244	12(63.2%)	23(40.4%)	10(41.7%)	0.208
	> 45	N (%)	18(64.3%)	37(51.4%)		7(36.8%)	34(59.6%)	14(58.3%)	
FH	-ve	N (%)	17(60.7%)	45(62.5%)	0.794	13(68.4%)	35(61.4%)	14(58.3%)	0.447
	+ve	N (%)	9(32.1%)	27(37.5%)		4(21.1%)	22(38.6%)	10(41.7%)	
ALT (U/L)		Median (range)	19(5–86)	19(3-199.9)	0.411	22(7–54)	19(5–86)	23.9 (3 199)	0.559
AST (U/L)		Median (range)	23(13-67.9)	23(9-147.1)	0.756	21(14.2–36)	23(11-67.9)	23.5(9-147.1)	0.740
T. bilirubin (mg/dL)		Median (range)	0.5(0.2-1)	0.5(0.2–1.2)	0.575	0.5(0.2-1)	0.5(0.2–1.2)	0.5(0.3-1)	0.610
ALP (U/L)		Median (range)	140(77–267)	137(66–355)	0.676	132(70–320)	137(66–355)	135.5(77–233)	0.908
Sr. Cr. (mg/dL)		Median (range)	0.8(0.6–2.6)	0.79(0.4–3.2)	0.273	0.8(0.4–1.1)	0.8(0.5–3.2)	0.8(0.4-2)	0.783
Uric acid (mg/dL)		Median (range)	4.2(3.3–11.5)	4.3(2.2–9.3)	0.531	4(2.8–5.6)	4.4(2.4–11.5)	4.3(2.2–9.3)	0.620
CEA (μg/L)		Median (range)	3.81(1–29)	2(0.5–33.5)	0.076	2.95(1–11)	2.74(1–29)	2(0.5–33.5)	0.474
CA15.3 (U/mL)		median(range)	22(9–82)	25(8.6–87)	0.260	22(8.6–82)	24(9–64)	27.2(14–87)	0.456
Albumin (mg/dL)		Mean ± SD	4 ± 0.6	4.1 ± 0.5	0.543	4.1 ± 0.6	4.1 ± 0.5	4 ± 0.6	0.893
WBCS (X10^9^/L)		Mean ± SD	7.4 ± 2.5	7.4 ± 2.5	0.901	7.3 ± 2.6	7.6 ± 2.2	6.9 ± 2.2	0.641
RBCS (cells/mc L)		Mean ± SD	4.5 ± 0.5	4.3 ± 0.6	0.063	4.2 ± 0.3	4.4 ± 0.7	4.2 ± 0.5	0.344
Hb (X10^9^/L)		Mean ± SD	12 ± 1.2	11.3 ± 1.9	0.097	11.6 ± 1.3	11.5 ± 2.1	11.4 ± 1.2	0.952
PLT (X10^9^/L)		Mean ± SD	250.6 ± 84.5	297.1 ± 80.1	0.094	296.8 ± 89.1	287.3 ± 89.7	266.5 ± 90	0.705

FH, family history; ALT, alanine transaminase; AST, aspartate transaminase; T. bilirubin, total bilirubin; ALP, alkaline phosphatase; SrCr, serum creatinine; CEA, carcinoembryonic antigen; CA15.3, cancer antigen 15 − 3; WBCS, white blood cells; RBCS, red blood cells; HB, hemoglobin; PLT, platelet; -ve, negative; +ve, positive.

**Table 6:** Association of rs4759314 A > G and rs920778 C > T genotypes with tumor features

			rs4759314			rs920778			
			**AA**	**AG**	**P-value**	**CC**	**CT**	**TT**	**P-value**
			**n = 28**	**n = 72**		**n = 19**	**n = 57**	**n = 24**	
Stages	**S1 + S2**	**N(%)**	26(92.9%)	61(84.7%)	0.342	17(89.5%)	50(87.7%)	20(83.3%)	0.818
	**S3 + S4**	**N(%)**	2(7.1%)	11(15.3%)		2(10.5%)	7(12.3%)	4(16.7%)	
	**S1**	**N(%)**	5(17.9%)	18(25%)	0.436	4(21.1%)	14(24.6%)	5(20.8%)	0.863
	**S2**	**N(%)**	21(75%)	43(59.7%)		13(68.4%)	36(63.2%)	15(62.5%)	
	**S3**	**N(%)**	1(3.6%)	8(11.1%)		2(10.5%)	4(7%)	3(12.5%)	
	**S4**	**N(%)**	1(3.6%)	3(4.2%)		0(0%)	3(5.3%)	1(4.2%)	
Grades	**G1 + G2**	**N(%)**	23(82.1%)	51(70.8%)	0.247	14(73.7%)	40(70.2%)	20(83.3%)	0.446
	**G3**	**N(%)**	5(17.9%)	21(29.2%)		5(26.3%)	17(29.8%)	4(16.7%)	
	**G1**	**N(%)**	6(21.4%)	16(22.2%)	0.443	3(15.8%)	11(19.3%)	8(33.3%)	0.531
	**G2**	**N(%)**	17(60.7%)	35(48.6%)		11(57.9%)	29(50.9%)	12(50%)	
	**G3**	**N(%)**	5(17.9%)	21(29.2%)		5(26.3%)	17(29.8%)	4(16.7%)	
ER/PR	**-ve**	**N(%)**	12(42.9%)	22(30.6%)	0.244	7(36.8%)	19(33.3%)	8(33.3%)	0.959
	**+ve**	**N(%)**	16(57.1%)	50(69.4%)		12(63.2%)	38(66.7%)	16(66.7%)	
*HER2*	**-ve**	**N(%)**	20(71.4%)	57(79.2%)	0.409	14(73.7%)	44(77.2%)	19(79.2%)	0.914
	**+ve**	**N(%)**	8(28.6%)	15(20.8%)		5(26.3%)	13(22.8%)	5(20.8%)	
Triple	-ve	**N(%)**	9(32.1%)	22(30.6%)	0.877	7(36.8%)	16(28.1%)	8(33.3%)	0.743
EP -ve / *Her2* + ve		**N(%)**	11(39.3%)	35(48.6%)	0.401	7(36.8%)	28(49.1%)	11(45.8%)	0.649
EP + ve / *Her2* –ve		**N(%)**	3(10.7%)	0(0%)	**0.004**	0(0%)	3(5.3%)	0(0%)	0.311
Triple	+ve	**N(%)**	5(17.9%)	15(20.8%)	0.826	5(26.3%)	10(17.5%)	5(20.8%)	0.705

ER /PR, estrogen/progesterone; *HER2*, human epidermal growth factor receptor 2; -ve, negative; +ve, positive.

### The rs4759314-rs920778 haplotypes’ association and risk for BC in the studied groups

A haplotype is a group of alleles inherited from a single parent. The rs4759314-rs920778 haplotypes’ statistical analysis showed that the AC haplotype reported the highest frequency among cases (34.8%), while AT showed the highest allele in controls (37.5%). The GC haplotype showed the lowest frequency among both groups. No association between haplotypes and the risk of BC was discovered (Table 7; Fig. 1b). The non-random connection of alleles at two or more loci in a population is referred to as “linkage disequilibrium” (LD). D′ can vary from 0 (no disequilibrium) to 1.

**Table 7:** Association of rs4759314 - rs920778 haplotypes with BC (maximum disequilibrium)

		Control	Cases	p-value	OR	95% CI	
		**Frequency**	**Frequency**				
rs4759314-rs920778	**AC**	0.325	0.348	**-**	**1**	**Reference**	
	**AT**	0.375	0.292	0.341	0.814	0.533	1.244
	**GT**	0.205	0.233	0.934	1.020	0.634	1.642
	**GC**	0.095	0.127	0.675	1.136	0.627	2.059

**Fig. 2:**
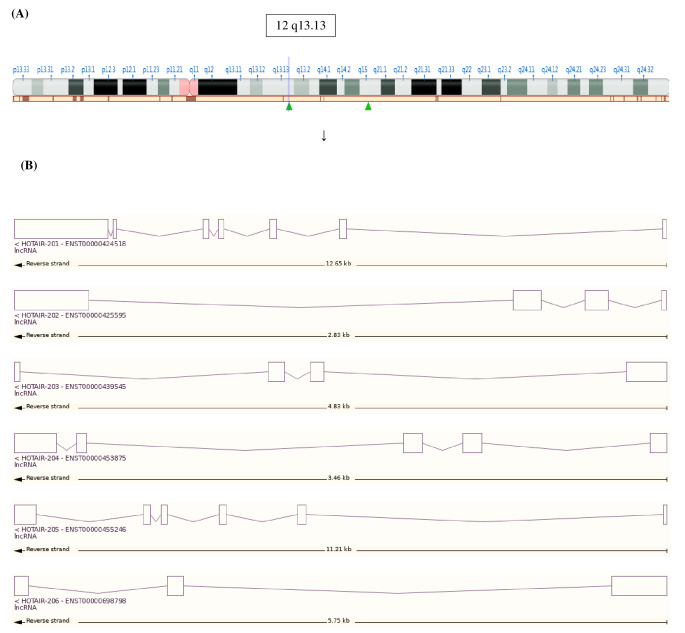
Genomic structure of the human *HOTAIR* gene. (**A**) Location of *HOTAIR* gene on chromosome 12q 13.13. The *HOTAIR* gene is located at chromosome 12q13.13 and transverses 12,649 nt (chr 12: (53,962,308.53,974,956) along the reverse strand. (**B**) The genomic structure of the *HOTAIR* transcripts. The *HOTAIR* gene consists of six splice variants, including *HOTAIR*-201, *HOTAIR*-202, *HOTAIR*-203, *HOTAIR*-204, *HOTAIR*-205, and *HOTAIR*-206, lncRNA with no protein-coding potential [Data source: NCBI database, Ensembl.org]

The bioinformatics of the *HOTAIR* gene is explained in Fig. 2. *HOTAIR* ENSG00000228630 was positioned at the long arm of chromosome 12q and spanned about 12 649 bases (Chr12: (53, 962, 308. 53, 974, 956) that were oriented with respect to the reverse strand. The *HOTAIR* gene comprises six splice variants based on its genomic structure (*HOTAIR*-201-206) (data source: Ensembl databases). The *HOTAIR* gene is a lncRNA. It has no protein-coding potential and is highly expressed in multiple tumors.

## Discussion

More than 80% of cancer-related SNPs have been identified in non-coding regions of the genome, according to genome-wide association studies. Most known lncRNAs are related to various cancer forms; however, their expression patterns are frequently specific to cell types and cancer types. One of the lncRNAs, *HOTAIR*, has been discovered as a BC risk factor and a biomarker for various malignancies [22]. Earlier research has shown that the expression of *HOTAIR* is considerably upregulated in both BC plasma and tissues. The detection of *HOTAIR* expression in plasma can be used instead of tissue biopsies as a biomarker for BC because it is a noninvasive technique with high sensitivity and specificity [23–25]. Among *HOTAIR* SNPs are rs920778 (C > T) and rs4759314 (A > G); Meanwhile, both were discovered to be related to higher expression of *HOTAIR*.

This study discovered that rs4759314 (A > G) was associated with an elevated BC risk in the heterozygote AG genotype, dominant, co-dominant, and overdominant models; however, there is no significant difference in rs920778 (C > T) genotype and allele frequencies, as well as no connection between *HOTAIR* (rs4759314, rs920078) variants and disease stages or histological grades.

Similarly, Minn et al. concluded that, in a Japanese population, the HOTAIR SNP rs920778 did not affect BC susceptibility. On the other hand, Lv et al. [26] discovered a strong relationship between rs920778 and rs4759314 and an elevated incidence of BC in the Northeastern Chinese population. A significant association between an enhanced risk of BC and the rs920778 polymorphism has been reported among Southeast Iranian ladies [27], the Turkish population [28], the Indian population [29], and Chinese cases [30]. Furthermore, Yan et al. [30] and Hassanzarei et al. [27] have investigated the link between rs4759314 and breast cancer susceptibility; however, their findings contradict the results in the current study. Contrary to our findings, Khorshidi et al. [31] investigated the association between three single nucleotide polymorphisms in the HOTAIR gene (rs12826786, rs1899663, and rs4759314). Regarding the prevalence of breast cancer in Iranians, they revealed that these polymorphisms do not appear to be associated with breast cancer risk. These discrepancies in results may be due to ethnic genetic diversity with different gene-gene interactions, gene-environment interactions, or probably due to other limiting factors related to sampling and the size of cases. The serum expression levels of HOTAIR, MALAT1, and NEAT1 were investigated in Egyptian patients by Abd El-Fattah et al. [32] using quantitative real-time PCR (qRT-PCR). They observed that the serum expression level of HOTAIR was significantly higher in the breast cancer patients compared to the fibroadenoma patients and the control subjects. Additionally, no other studies link these two SNPs to cancer among Egyptians.

According to prior research on other diseases, the allelic frequencies of the *HOTAIR* SNPs rs12826786 and rs920778 were not statistically different between cancer-free controls and glioma patients [33]. Oliveira et al. [34] showed that rs12826786 and rs920778 are not significantly correlated with prostate cancer susceptibility among Portuguese. Kim et al. [35] tested the correlation between colorectal cancer susceptibility and *HOTAIR* variants; however, they showed no association between rs920778, rs4759314, and breast cancer among the Korean population. This may reflect the fact that a population’s susceptibility to a disease may vary depending on the cancer type and the individual’s gender [36].

Based on a meta-analysis that investigated the connection between *HOTAIR* polymorphisms and risks of BC, cervical cancer, and ovarian cancer, only rs4759314 was substantially correlated to a lower risk of BC, ovarian cancer, and cervical cancer. At the same time, rs920778 and rs18995663 were linked to breast, cervical, and ovarian cancer [37]. By meta-analysis, Liu et al. [15] found a link between overall cancer risk and rs920778 and rs4759314 polymorphisms. Other meta-analyses revealed the contribution of the *HOTAIR* rs920778 mutation to the elevated cancer risk, but rs4759314 had no significant connection [38, 39]. Another meta-analysis found no difference between *HOTAIR* rs920778 and rs4759314 in relation to breast cancer susceptibility [40, 41]. A meta-analysis conducted by Wang et al. [6] showed a strong link between *HOTAIR* rs920778 and the BC risk, but there was no strong link between the rs4759314 polymorphism and the BC risk.

There is a low distribution frequency of the uncommon genotype GG of rs4759314 among patients (0%) and controls (5%); therefore, evaluating their relationship with BC requires a larger sample size. According to our knowledge, this is the first study to examine the association between these two polymorphisms and BC among Egyptians.

## Conclusion

Susceptibility and illness progression differ from one community to the next due to gene-gene and gene-environment interactions; thus, gene expression could be population-specific. Therefore, the results of this study explored that rs4759314 (A > G) could be a BC risk factor among Egyptian women, and patients with ER/PR positivity and *HER2* negativity were significantly associated with the AA genotype compared to the AG genotypes. However, larger case-control research should be recommended to evaluate the impact of *HOTAIR* SNPs on BC and measure *HOTAIR* levels in plasma.

## Data Availability

The datasets generated and/or analyzed during the current study are available in the manuscript.
